# The Effect of Hive Type on Colony Homeostasis and Performance in the Honey Bee (*Apis mellifera*)

**DOI:** 10.3390/insects15100800

**Published:** 2024-10-14

**Authors:** Rola Kutby, Barbara Baer-Imhoof, Samuel Robinson, Lucy Porter, Boris Baer

**Affiliations:** 1 Center for Integrative Bee Research (CIBER), Department of Entomology, University of California Riverside, Riverside, CA 92521, USA; rolakutbi@hotmail.com (R.K.); barbarai@ucr.edu (B.B.-I.); 2 School of Molecular and Life Sciences (MLS), Curtin University, Kent St., Bentley, WA 6102, Australia; 3 Ducks Unlimited Canada, Stonewall, MB R0C 2Z0, Canada; samuel.robinson@ucalgary.ca; 4 Center for Integrative Bee Research (CIBER), Bayliss Building (M316), The University of Western Australia, 35 Stirling Highway, Crawley, WA 6009, Australia; lucykporter@gmail.com

**Keywords:** Langstroth, Warré, abiotic stress, temperature, humidity, bee health, immunocompetence

## Abstract

**Simple Summary:**

We provided honey bees (*Apis mellifera*) with two different hive setups and compared the bees’ ability to maintain colony homeostasis. We conducted a field experiment in a Mediterranean climate covering the period when honey bees were most active, including the dry and hot summer months. We found that bees were perfectly capable of maintaining temperature and humidity within remarkably narrow ranges in areas where vulnerable individuals are present, such as frames with developing brood and the queen, but that the hive setup impacted the bee’s ability to regulate them. We conclude that the hive type and setup can provide opportunities to manage honey bee health, which could be helpful in the future to mitigate the effects of climate change.

**Abstract:**

The colonies of honey bees are mostly sessile organisms. Consequently, the type of nest boxes that beekeepers provide to their bees should impact a colony’s ability to maintain homeostasis, which is a key determinant of performance and fitness. Here, we used European honey bees (*Apis mellifera*) and provided them with two hive setups widely used and known as Langstroth and Warré. We compared colony performance in a Mediterranean climate for five months from late spring to early autumn, which covered the most active time of bees and included periods of heat and drought. We found that irrespective of hive type or season, honey bees kept hive temperature and humidity within a remarkably narrow range. Nevertheless, the hive type impacted the daily fluctuations in temperature and humidity. In Warré hives, where bees have more autonomy to build and maintain their combs, we found that bees were able to reduce daily fluctuations in temperature and humidity and kept both measures closer to the overall average. This increase in colony homeostasis found in Warré hives negatively correlated with other hive performance indicators, such as immunocompetence. We conclude that different hive types affect key areas, such as the central part of the colony with frames of developing brood or the queen, which are the most susceptible individuals. This implies that climatic changes resulting in extreme weather events are expected to impact colony performance and fitness, especially in non-managed honey bees that are limited by available nesting sites. For managed bees, adaptations to existing hive setups could be provided to help bees minimize the effects of abiotic stress.

## 1. Introduction

Recent declines in honey bee populations are very concerning, given their ecological and economic importance. Significant hive losses of managed honey bee hives have been reported on a global scale [1,2], and beekeepers in the US lost almost half of their managed honey bee colonies in 2023 [3]. Researchers identified multiple environmental factors that can negatively impact bees, including parasites and pathogens [4,5,6], the exposure of bees to pesticides in agricultural and urban landscapes [7,8], habitat loss [9,10], and climate change [11,12].

1. Hive setups used to keep honey bees

Humans have domesticated honey bees for thousands of years [13,14]. This process included developing structures to house and transport bees, which is documented as far back as ancient Egypt [15,16]. Over time, bee husbandry increasingly diverged from the way bees naturally build and maintain their colonies. The industrial and agricultural revolution further increased the demand for bees and their pollination services and required beekeeping operations to maximize economic profitability. Lorenzo Lorraine Langstroth developed a beekeeping setup in 1852, which is used widely today [17,18]. Langstroth hives provide bees with frames that contain a plastic or wax foundation to encourage honey bees to build their wax combs. The frames are kept in wooden boxes of a standardized size of up to 10 frames, allowing beekeepers easy access to inspect hives for diseases and to remove, add, or change frames as colonies increase or decrease in size throughout the season [17,19]. A brood box at the bottom of the hive is often separated from other areas of the hive used for honey storage using a queen excluder, which restricts the egg-laying of queens to certain areas of the hive. Because of these advantages, the Langstroth hive setup is used for commercial beekeeping [20,21]. At the beginning of the 19th century, Abbé Émile Warré developed a different hive setup [22]. Warré hives provide bees with only a wooden bar on the top of a wooden nest box that allows bees to build their wax combs. The queen is not restricted to certain hive areas; therefore, the brood can be reared in any part of the hive. Because the resulting combs vary in size and shape between frames, they are not easily removable or interchangeable, making honey extraction more difficult. However, allowing bees to freely manage their comb setup represents a more natural situation and was hypothesized to benefit the bee’s ability to maintain colony homeostasis [23]. This should be particularly advantageous for colonies experiencing abiotic stress, such as periods of heat or drought [24,25], which has become increasingly important because of global climate change.

2. Temperature as a key factor of colony homeostasis

Honey bees regulate the temperature inside their hives between 32 °C and 36 °C [26,27,28,29,30]. Typical hive temperatures reported in the literature vary, however, and they seem to be influenced by geography or the specific location where the temperature was measured inside the hive. Thermoregulation is known to incur significant costs for honey bees [31,32,33], and bees minimize them by closing cavities and manipulating the size of the hive entrance [34,35] with wax or propolis to regulate airflow through the hive. To avoid heat loss during cold weather, worker bees form dense clusters and generate heat by shivering [36,37], which is a behavior where individuals move their flight muscles. The heat produced by these bees is transferred to the combs and brood by wing beats [28,38]. Bees on the periphery of the hive can act as insulators to avoid both heat loss and overheating [39,40]. For the latter, bees evaporate water collected outside by beating their wings [41,42,43] as a form of evaporative cooling. To increase hive ventilation, workers assemble in clusters outside the hive, which is a behavior known as bearding [44,45].

The regulation of hive temperature is crucial for developing brood, which is sensitive to small temperature changes [46,47]. Honey bee eggs and pupae exposed to temperatures below 20 °C experience cold stress, which increases mortality [48], prolongs development time [49], and reduces adult longevity [48], as well as short-term learning and memory [50]. Workers reared at temperatures above 36 °C or below 32 °C can develop deformed brains, wings, stingers, proboscis or legs [51] and are more susceptible to insecticides [52]. Because the viscosity of bee wax is affected by the surrounding temperature, hive temperatures affect the bees’ ability to build combs [53]. Heat stress reduces the fertility of queens and males [54,55]. Male larvae exposed to heat have smaller reproductive organs and reduced sperm viability [55]. In adult males, exposure to increased temperature exposures of 1–3 °C reduces sperm viability in the ejaculate [56].

3. Humidity as a key factor of colony homeostasis

Honey bees also regulate the humidity inside their hives, which ranges between 40 and 95% [57,58]. Humidity is higher in larger colonies, and hives where more developing brood frames are present [58]. Hive humidity increases when bees lower hive temperatures by evaporating water (see above [42,43]) or during honey production when bees evaporate water to reduce the water content of collected nectar to ~16% [59]. Honey bees can reduce hive humidity through the fanning behavior described above [41] to replace more humid air with drier air from the environment [31]. Humidity is critical for the development of the brood [60,61,62] and, therefore, fluctuates less in areas where worker brood is present compared to male brood [63]. Elevated hive humidity reduces infestations of parasitic mites (*Varroa destructor*) [64] but also increases the risk of nectar fermentation [65].

4. Colony Homeostasis and Immunocompetence

Immune defenses are costly for hosts, both energetically as well as physiologically, because immune effector molecules can also harm the host as a form of autoimmunity. These costs are also expected to trade off with other life-history traits, including colony homeostasis. In the case of honey bees, we can expect that individual bees and larvae living in hive setups that support their efforts to maintain stable hive conditions experience less stress and lower the risk of spreading diseases. Such environments can then allow individuals to reduce their investments into costly and potentially damaging immune defenses. Consequently, quantifying colony immunocompetence provides an opportunity to test this idea and we predicted that bees kept in Warré hives show reduced investments into immunocompetence.

Given the well-documented importance of temperature and humidity for honey bees, we compared colony homeostasis and the performance of colonies we kept in Langstroth and Warré hives. To do this, we conducted a controlled field experiment for 130 days covering early spring to autumn, when bee hives are most active and experience climatic stressors such as periods of heat and drought [24]. We predicted that colonies in Warré hives are better able to manage these environmental stressors, resulting in increased colony performance and immunocompetence compared to bees kept in Langstroth hives.

## 2. Materials and Methods

### 2.1. Colony Setup

We purchased four standard Warré- and four 8-frame Langstroth hive setups from commercial vendors in Western Australia and set them up in our apiary at the Center for Integrative Bee Research (University of Western Australia) in Perth. To minimize the potential effects of drifting bees, we (1) painted geometrical forms of different shapes and colors above the entrances of hives, (2) increased the distance between them to approximately 2 m, and (3) arranged the hives in 2 separate lines, where the hive type was alternated between Langstroth and Warré. We kept all hives in the same location under a shading cloth and provided each hive with 225 g of sugar syrup. We purchased eight packages of live bees and used a commercial TPW beekeeping scale to weigh each package. We calculated the weight of the live bees by subtracting the weight of the empty box from the total weight of the package. We calculated the mean weight of live bees per package, which was 1.87 ± 0.05 kg (mean ± SEM) and comparable between the different packages. This ensured that all hives started with approximately the same number of worker bees. At the start of the experiment (day 0), we provided each hive with frames (Langstroth) or bars (Warré) and introduced a single, newly mated queen. These queens originated from a single mother and were provided by the Western Australian queen breeding program Better Bees. We inspected the hives on day 6 of the experiment to confirm that the queen had started to lay eggs and that larvae were present. Throughout the experiment, we provided colonies with additional space as needed, such as extra boxes or frames. One of the Warré colonies replaced their queen during the experiment with a self-raised, openly mated daughter queen, which we left in the hive until the end of the experiment. The experiment ran for a total of 130 days from the 9 October 2015 (day 1) to the 16 February 2016 (day 130).

### 2.2. Colony Homeostasis and Performance

On day 28 after setting up the experimental hives, we inspected them to confirm that they were fully established, as indicated by the presence of an egg-laying queen, all stages of a developing brood, and stored honey and pollen. We then identified the location of the core brood area to place commercially available EL-USB-2-LCD data loggers (URL accessed on 1 October 2024: https://www.lascarelectronics.com/easylog-el-usb-2-lcd) between two brood frames as close as possible to the developing brood. Therefore, we collected data from the hive area where individuals most susceptible to climatic stress were present and where we predicted bees to display maximal efforts to regulate temperature and humidity. We subsequently collected temperature and humidity data every 20 min for 71 days. To prevent bees from covering the loggers with propolis or wax, we wrapped each device in a fiberglass gauze, which we inserted into a 50 mL centrifuge tube without a lid. At the end of the experiment, we retrieved all sensors and downloaded data to a laptop computer using EasyLog USB software version 7.7 (URL accessed on 1 October 2024: https://lascarelectronics.com/software/easylog-software/easylog-usb/).

For statistical analyses, we used temperature and humidity data recorded at 9:00 a.m. when colonies had fully established their daily activities as well as at 3:00 p.m. when climatic stressors were expected to be maximal. We downloaded corresponding meteorological data from the nearest weather station of the Australian Bureau of Meteorology in Swanbourne (Station # 009215), 8.7 km from the colonies’ location, and used them as a reference for the environmental climatic conditions.

We weighed each colony on day 1 (the start of the experiment), day 55 (corresponding to spring and the deployment of the sensors), day 82 (summer), and day 130 (late summer and the end of the experiment). To carry this out, we placed the hive on an electronic scale and recorded the total weight to the nearest gram. We calculated weight gains or losses and corrected for any hive components we had added or removed (see above). To quantify immunocompetence in worker bees, we measured encapsulation response as described in [66]. In short, we collected 10 foraging workers, which were individuals returning to their colony from flights, and collected them at the hive entrance during days 13–15 of the experiment as well as between days 110 and 113, resulting in a total sample size of 160 bees. We anesthetized each worker with CO_2_ and placed them into a modified apparatus normally used to artificially inseminate honey bee queens (URL accessed on 1 October 2024: http://bee-insemination.com/). Using a fine, alcohol-sterilized injection needle, we punctured a small hole into the intersegmental membrane between the second and third sternite. Next, we used INOX watchmaker forceps and inserted a 2 mm × 0.5 mm nylon piece into the hemolymph of the bee. Individuals were allowed to recover, transferred into boxes separated by hive origin, and fed with sugar water ad libitum. We kept bees in an incubator at 33 °C for 24 h before freeze-killing them and dissecting their abdomens to remove the implants. Each implant was transferred to a droplet of Eukitt’s quick-hardening mounting medium (URL accessed on 1 October 2024: https://www.sigmaaldrich.com/SA/en/product/sial/03989) on a microscopic slide and covered with a cover slip. Using a digital Canon EOS 5 camera, we took pictures of each implant and quantified the degree of melanization as the difference in gray value between the implant and the slide background using the software Image J 2.0.0-rc-30 (URL accessed on 1 October 2024: https://imagej.net/ij/download.html).

To measure colony performance, we quantified the comb area containing honey, pollen, or brood at the start of the experiment on day 16 and the last day of the experiment on day 130. To carry this out, we gently removed all bees from a frame with a bee brush and took a digital photo of each side. We used the software Image J to measure the total comb area [67] as well as the areas containing capped brood, pollen, or honey.

### 2.3. Data Analysis

To statistically compare temperature and humidity data, we used linear mixed-effects models in R (R Core Team 2022) using the lme4 package [68]. For models of variables, we measured daily temperature and humidity, hive type (Langstroth and Warré), time of day (AM and PM), and their interactions as fixed effects. Date and colony ID were included as random intercepts in all models. To analyze hive performance (encapsulation, hive weight, wax comb area, honey-filled comb), we used hive type (Langstroth or Warré), season (spring and late summer), and their interactions as fixed effects. For the colony weight measurements, we calculated changes in weight concerning our initial measurements, resulting in time being a factor with three levels (spring, summer, and late summer). Residuals and random intercepts were visually checked for normality and equal variance. To meet the normality assumptions for ANOVA, we square root transformed the overall comb variable and log-transformed the honeycomb variable. We considered *p*-values to be significant at the 0.05 level.

## 3. Results

### 3.1. Temperature

**Figure 1:**
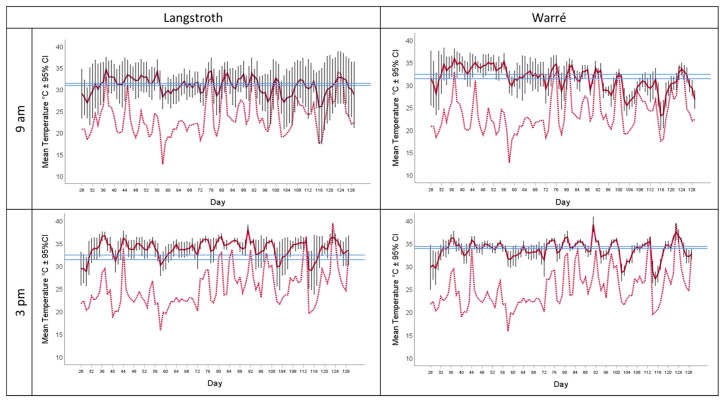
The solid red lines provide mean hive temperatures during the experiment in °C ± 95% CI, measured in the brood area of either Langstroth (**left panels**) or Warré (**right panels**) hives. The corresponding outside temperatures are provided as dotted red lines. Temperatures recorded at 9:00 a.m. are shown in the upper and those collected at 3:00 p.m. in the lower panels. The blue bar provides the corresponding medians ± 95% CI calculated for each hive type and time of the day.

We found that all experimental hives regulated the temperature inside their hives within a very narrow range, irrespective of hive type or season. We found a median ± 95% CI hive temperature of 33.50 [33.00, 33.50] °C (Table 1, Figure 1). The corresponding environmental temperatures ranged from 12.80 °C to 34.70 °C in the mornings and 15.90 °C to 39.50 °C in the afternoons, indicating that colonies experienced periods of cold and heat stress during the experiment and during times when bees were active.

**Figure 2:**
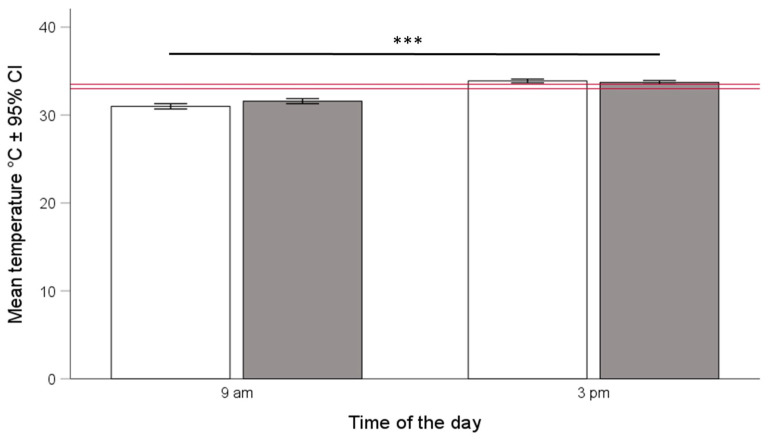
Mean hive temperatures in °C (±95% CI) at 9:00 a.m. and 3:00 p.m. for honey bee colonies kept in Langstroth (white bars) and Warré (grey bars) hives. The horizontal red bar shows the overall median ± 95% CI. Mean temperatures differed between hive types as well as the time of the day as indicated by a significant hive type × time of the day interaction term (ANOVA, hive type × time of day interaction, F_1, 1521_ = 22.79, *p* < 0.001). The three asterisks (***) indicate levels of significance between groups where *p* values are < 0.001.

Hive temperatures were correlated with environmental temperatures although correlation coefficients were low (overall Pearson correlation, r = 0.23), both in the mornings (r = 0.35) and afternoons (r = 0.14). Hive temperatures were consistently higher compared to the environmental ones, and this was the case in the mornings when the hive temperature was 7.45 °C ± 0.29 (mean ± 95% CI) higher, as well as in the afternoons (8.47 °C ± 0.24, mean ± 95% CI). Compared to Langstroth hives, temperatures in Warré hives were significantly higher in the mornings but lower in the afternoons (Table 1, Figure 1). The honey bees we kept in the Warré hives kept temperatures closer to the calculated overall range (see the red bar in Figure 2, ANOVA, hive type × time of day interaction, F_1, 1521_ = 22.79, *p* < 0.001).

### 3.2. Humidity

#### 3.2.1. Relative Humidity (RH)

**Figure 3:**
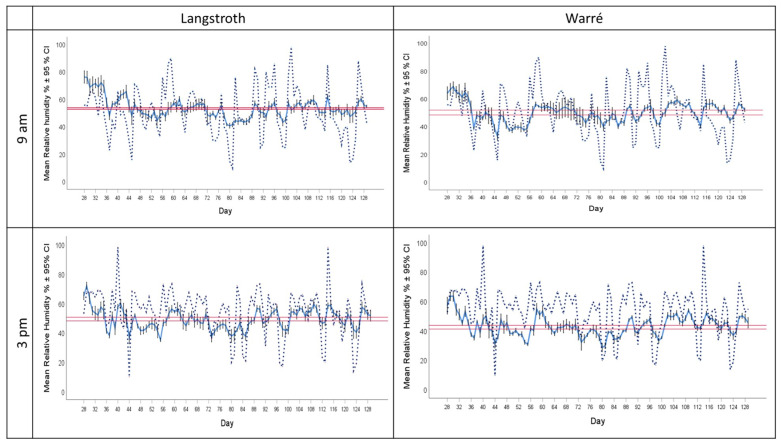
The solid blue lines show relative humidity (RH) (mean ± 95% CI) of honey bee colonies kept in Langstroth (**left panels**) and Warré (**right panels**) hives in the mornings (**upper panels**) and afternoons (**lower panels**). Corresponding outside RH is provided by the dotted blue lines. The horizontal red bars show the overall median ± 95% CI for each hive type and time of the day.

**Figure 4:**
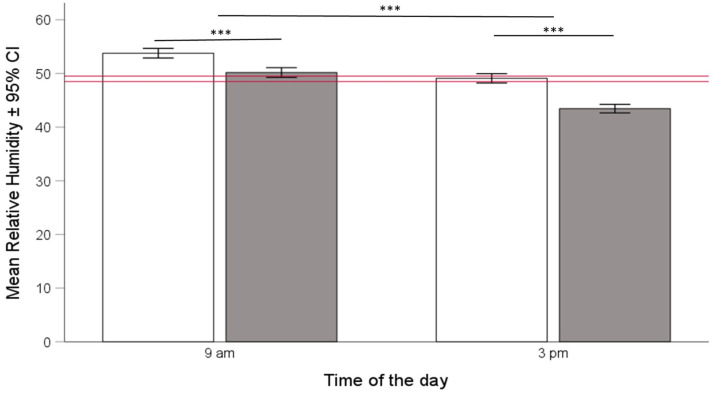
Mean relative humidity (RH) in % ± 95% CI in honey bee hives at 9:00 a.m. and 3:00 p.m. in Langstroth (white bars) and Warré (grey bars) hives. The horizontal red bar shows the overall median ± 95% CI. Mean relative humidity was lower in Warré hives, but the difference was larger in the afternoons compared to the mornings (ANOVA, hive type × time of day interaction, F_1, 1521_ = 12.36, *p* < 0.001. The three asterisks (***) indicate levels of significance between groups where *p* values are < 0.001.

We found that the overall hive RH was 49.0 ± 0.50% (median ± 95% CI). Similar to temperature, RH correlated with environmental RH (overall Pearson correlation, r = 0.31) both in the mornings, r = 0.32, and in the afternoons, r = 0.41. Hive RH was significantly lower compared to the corresponding environmental RH (Figure 3, paired T-test, df = 1632; t = 7.78; *p* < 0.001). RH was significantly higher in Langstroth compared to Warré hives, and this difference was larger in the afternoons compared to the mornings, as indicated by a significant hive type × time of day interaction term (Figure 4, ANOVA, hive type × time of day interaction, F_1, 1521_ = 12.36, *p* < 0.001).

#### 3.2.2. Absolute Humidity (AH)

RH is dependent on the temperature, and this impacted our data because hive temperatures were substantially (i.e., 7–8 °C, see above) higher compared to the corresponding environmental ones (Figure 1). To address this issue, we calculated absolute humidity (AH) using the following equation [69]:

$$\mathrm{AH}\;\left({\mathrm{Absolute}\;\mathrm{Humidity},\;\mathrm{grams}/m}^{3}\right)=\frac{6.112\times e^{\left[\left(17.67\times \;T\right)/\left(T\;+243.5\right)\right]\;}\times \;\mathrm{RH}\;\times 2.1674}{273.15+\;T},$$

where T is the temperature in °C and RH is the relative humidity in %.

**Figure 5:**
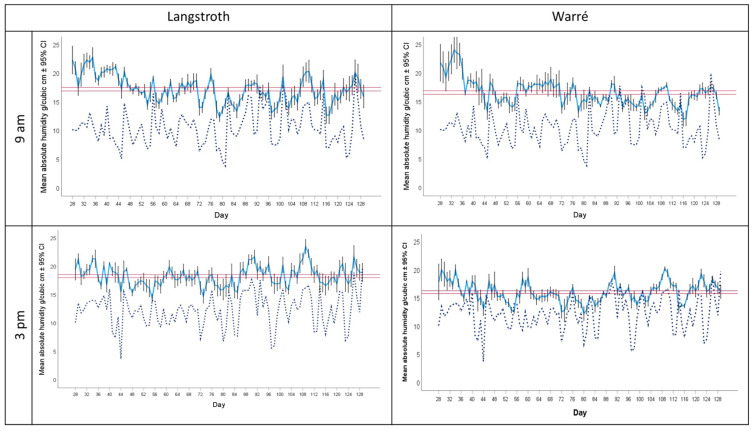
Blue solid lines show mean ± 95% CI absolute humidity (AH) in Langstroth (**left panels**) and Warre (**right panels**) hives; the corresponding outside AH is provided by the dotted blue line. Upper panels show morning data at 9:00 a.m. and lower panels at 3:00 p.m. The horizontal red bar represent the corresponding median ± 95% CI calculated for each hive type and time of the day separately.

The mean AH in all hives was 17.07 ± 0.15 g/m^3^ (±95% CI) and varied significantly less compared to environmental AH (paired T-test, df = 1631, t = 60.48, *p* < 0.001). Hive and environmental AH were moderately correlated (Pearson correlation, r = 0.27 for all data, r = 0.23 for the mornings, and r = 0.32 for the afternoons). Hive AH was consistently higher compared to environmental AH and significantly higher in the mornings compared to the afternoons (paired T-test, df = 815, t = −2.39, *p* < 0.05), (Figure 5), indicating that the presence of the bees and their activities increase AH inside their hives.

**Figure 6:**
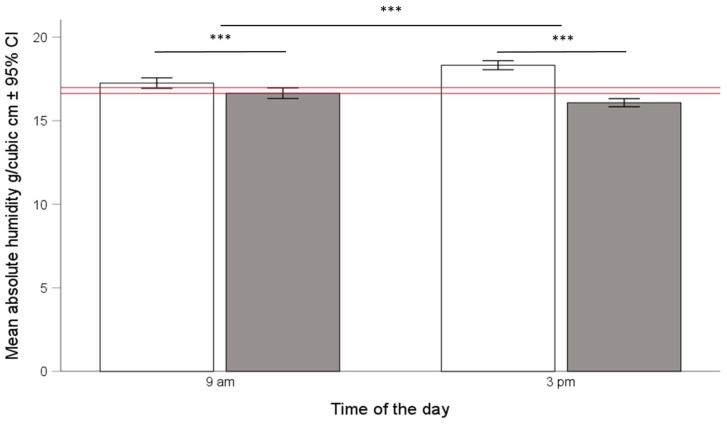
Mean absolute humidity (AH) ± 95% confidence interval in the brood area of Langstroth (white bars) and Warré hives (grey bars) in the morning (**left side**) and afternoon (**right side**). The horizontal red bar represent the overall median ± 95% CI. Mean absolute humidity was lower in Warre hives, but the difference was larger in the afternoons compared to the mornings (ANOVA, Hive type × Time of the day interaction, F_1, 1521_ = 50.02, *p* < 0.001). The three asterisks (***) indicate levels of significance between groups where *p* values are < 0.001.

AH was consistently higher in Langstroth compared to Warré hives (Figure 6) and increased in Langstroth hives in the afternoon but decreased in Warré hives, as indicated by a significant hive type × time of day interaction term (ANOVA, F_1, 1521_ = 50.02, *p* < 0.001).

### 3.3. Colony Performance

**Figure 7:**
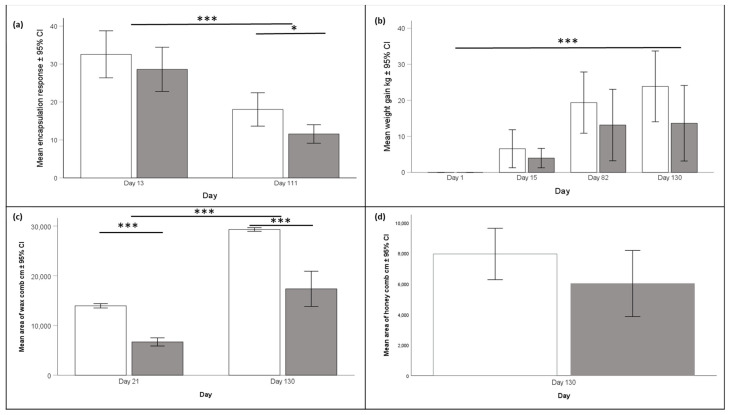
The white bars represent data collected from Langstroth hives, and the gray bars represent data from Warré hives. (**a**) Mean ± 95% CI encapsulation response of bees was higher at the start than at the end of the experiment (ANOVA, F_1, 156_ = 41.74, *p* < 0.001) and higher in bees collected from Langstroth-compared to Warré hives. The latter was significant for the bees we collected at the end of the experiment (ANOVA, F_2, 12_ = 39.28, *p* < 0.05). (**b**) All colonies gained weight during the experiment irrespectively of hive type (ANOVA, F_2, 12_ = 39.28, *p* < 0.001. (**c**) The mean area of wax comb in Langstroth and Warré hives was significantly higher at the end (Day 130) of the experiment compared to the start (ANOVA, F_1, 6_ = 129.62, *p* < 0.001). The increase in wax comb area differed between the two hive types (ANOVA, F_1, 6_ = 53.94, *p* < 0.001. (**d**) Langstroth hives stored more honey at the end of the experiment compared to Warré hives but the difference was not statistically significant. A single asterisk (*) indicates statical differences where *p* < 0.05 while three asterixis (***) are used to indicate *p* values < 0.001.

Encapsulation response was significantly higher in workers that we sampled from Langstroth hives compared to bees originating from Warré hives (Figure 7a, ANOVA, F_1, 156_ = 39.28, *p* < 0.05). Furthermore, encapsulation responses were significantly lower at the end of the experiment compared to the beginning (ANOVA, F_1, 156_ = 41.74, *p* < 0.001) (Figure 7a). Colonies kept in Langstroth hives gained significantly more weight during the experiment compared to those in Warré hives (Figure 7b, ANOVA, F_2, 12_ = 39.28, *p* < 0.001). Total comb area increased significantly during the experiment in all hives (Figure 7c, ANOVA, F_1,6_ = 129.62, *p* < 0.001), and this was the case for both hive types (Figure 7c, ANOVA, F_1,6_ = 53.94, *p* < 0.001). Hives also increased the amount of stored honey during the experiment (Figure 7d, ANOVA, F_1,6_ = 697.44, *p* < 0.0001), but we did not find any significant difference between Langstroth and Warré hives. We found the same statistical results for comb area containing brood or pollen, which both increased from the start to the end of the experiment, but these increases did not differ between Langstroth and Warré hives.

## 4. Discussion

We provided honey bees with two hive types and tested whether this impacted their ability to maintain hive temperature and humidity, which are both key determinants of brood-rearing success [57,58,59,60,61], colony performance [46,70] and drone/queen fecundity [55,71]. We conducted a field-based experiment in a Mediterranean climate covering the seasons of maximal hive activity between spring and the end of summer. To our knowledge, our dataset provides the longest period of uninterrupted and simultaneous temperature and humidity recordings in two different bee hive types. All colonies survived to the end of the experiment and increased their comb areas and food storage, indicating that they successfully progressed as expected despite periods of hot and dry climatic conditions (Figure 1 and Figure 5). Our experiment provides empirical support for the idea that bees in Warré hives are indeed better able to maintain colony homeostasis, as indicated by our finding that they kept hives closer to the overall ranges of temperature and humidity. However, our data offer a number of interesting new insights into the way honey bees manage colony homeostasis and the influence of external abiotic factors, such as hive types, time of the day, or climate. In the paragraphs below, we will discuss them and put them into a broader context of bee biology and beekeeping.

### 4.1. Abiotic Stress in Honey Bee Hives

The maximal temperature we measured in the brood area of our hives was 40.50 °C, indicating that colonies experienced heat stress during some periods. Although environmental temperatures dropped significantly below the typical range of hive temperatures provided in the literature, we did not find any indications of cold stress, given that hive temperatures never dropped below 19 °C (Table 1). Similarly, absolute humidity measures were consistently lower in the environment compared to those present inside the hives, implying that bees continuously increased humidity. In addition to comparing hive temperature and humidity with those present in the environment, we also analyzed our hive data over time and found that honey bees maintained temperature and humidity within remarkably narrow ranges throughout the experiment, and they fluctuated much less compared to the climatic conditions present in their environment. Furthermore, although we found statistical effects between hive types or the time of the day, the absolute differences were small. (Figure 1 and Figure 5). Therefore, we decided to calculate overall medians ± 95% confidence intervals for hive temperature and humidity and defined them as the optimal hive ranges. We used medians instead of means to discriminate against possible outliers, for example when colonies experienced abiotic stress conditions. This allowed us to define climatic stress as events when we found measurements to be outside of these confident intervals of temperature and humidity. As seen in Figure 1 and Figure 5, this occurred multiple times throughout our experiment, providing additional and independent support for the idea that hives indeed experienced climatic stress during some periods that required them to invest energy for cooling or heating to maintain colony homeostasis. Furthermore, calculating and comparing ranges and variations in temperature and humidity rather than means might provide better measures of colony homeostasis to identify colonies that are experiencing abiotic stress. Deviations from such narrow ranges of temperature and humidity are known to have a range of negative effects on colony performance. Consequently, the monitoring of temperature and humidity in the case of honey bees could be used in the future to identify early signs of declining health or indicate possible collapses. Such an approach was recently used using hive temperature data (Hossain et al., 2024, [72]), enabling the development of the first algorithm that is able to predict future hive performance and the possibility of triggering alarms of declining hive health at a time when colony collapse can be avoided.

### 4.2. Colony Homeostasis in Warré Hives

Calculating optimal temperature and humidity ranges also allowed us to test for the influence of hive type on the bees’ ability to maintain colony homeostasis. We found support for the idea that the honey bees we kept in Warré hives were indeed better able to maintain colony homeostasis because temperatures and humidities in Warre hives were, on average, closer to the overall ranges (See Figure 2, Figure 4, and Figure 6), and this was the case in the mornings and afternoons. Warré hives are designed to provide better ventilation compared to Langstroth hives and contain wood shavings that act as a humidity sink. This implies that relatively small modifications to hive boxes used to keep bees impact the central part of a hive where individuals most susceptible to temperature and humidity changes are present. Although the absolute differences in the ranges were comparatively small (see bars provided separately for hive types and times of the day in the different panels of Figure 1 and Figure 5), the energetic investments in achieving this might have differed, something that should be studied in more detail in the future.

For example, we found that Warre hives gained less weight and stored less honey at the end of our experiment. The reason for this difference in weight gain could have been a founder effect, given that bees in the Warré hives had to produce additional wax to build their new combs, whereas the bees in the Langstroth hives were provided with frames containing a plastic foundation. The production of wax for comb building is known to be energetically demanding because it takes honey bees between 6.6 and 8.8 kg of honey to produce 1 kg of bee wax [73,74]. It would have been interesting to know whether this would have impacted winter survival, or whether hive performance would have been different during a second season, given Warré hives would then have started with fully drawn combs.

### 4.3. Immunocompetence Differs between Hive Types

We found that worker immunocompetence differed between the two hive types and was—as predicted—lower in Warré hives compared to Langstroth hives. Immunocompetence increases at the start of adult life in honey bee workers and then declines with increasing age as a form of immune senescence [75]. To ensure that we sampled mature individuals, we collected foraging bees because these bees are at least 10–12 days old. Because we analyzed and interpreted our data in a comparative way such as hive types, our results cannot be explained by age-related variations within our sampling population. Despite this variation, we found that workers returning to Langstroth hives had a stronger encapsulation response than those returning to Warré hives. Our measure of encapsulation response quantifies an individual bee’s response to a foreign object inside its body and was significantly higher at the start of the experiment compared to the end. This can be explained by an increased investment in immunocompetence and a higher risk of becoming infected with parasites in spring, for example, through the shared use of flowers [76] and during times of food abundance and maximal foraging activity. Given that we did not observe any major disease outbreaks in any of our colonies and the absence of several virulent honey bee pests, such as *Varroa*, *Nosema ceranae*, several viruses, or the small hive beetle [77], it is possible that the Warré hive setup allowed bees to avoid or reduce overall parasitism loads and individual bees therefore reduced their energetic investments into costly immunocompetence. This could be easily tested in the future by comparing disease levels and propagation in the two hive types. Determining the causal link between hive type and parasitism and immunocompetence was beyond the scope of this work, but it would be important for future bee health management.

### 4.4. Absolute Humidity as a Variable of Colony Homeostasis

Previous research published reported values of relative humidity (RH) inside honey bee hives [58,59,60,61,64,65]. The overall mean hive RH in our dataset was 49.1 ± 0.47% (95% CI) and, therefore, higher compared to the 40% reported by Human et al. (2006) [57] but substantially lower compared to the 90–95% mentioned by Doull, (1976) [58]. We found that relative humidity was substantially lower inside honey bee hives compared to the corresponding measurements we obtained from the environment. This was difficult to explain because we were not aware of any mechanism that would allow honey bees to actively dehumidify air. Therefore, we calculated absolute humidity, which considered the substantial temperature differences we found between hives and their environment. Overall, the bees in the Warré hives maintained AH closer to the overall ranges we calculated, indicating bees in Warré hives were also able to better maintain humidity compared to bees in Langstroth hives. We found that—as expected—absolute humidity was substantially higher in the hives compared to the environment, confirming that the presence of bees and their activities increased humidity. Like temperature, variation in hive AH was substantially lower compared to AH in the environment, implying that bees actively kept and maintained AH within preferred ranges by increasing AH through evaporating water or reducing it through fanning (see introduction). We conclude that absolute humidity (AH) might be a better measure to describe colony homeostasis and could be used in the future as a measure of identifying colonies experiencing climatic stress or declining health.

### 4.5. Hive Temperature Cycles

We found that hive temperatures were several degrees higher compared to environmental temperatures, even during the hot Australian summer. This was the case in the mornings and afternoons (Figure 1). The higher hive temperatures we consistently measured in the afternoons can therefore not be explained by environmental fluctuations in temperature. This implies that the in-hive temperature cycling is regulated by the bees and that inside and outside temperatures are not causally linked, as already indicated by the low correlation coefficients. Our findings support earlier research that showed that in-hive temperatures are not constant [78,79] and could explain the broader range of hive temperatures that have been reported in the literature [27,30,71]. The reasons for these temperature cycles remain to be studied. Higher in-hive temperatures in the afternoons could be caused by increased activities when foragers return to their hives [80] and the subsequent processing of food and brood care (for example, feeding). Alternatively, honey bees could deliberately alter hive temperatures to maximize hive performance. Because bees are exothermal animals, environmental temperatures determine their physiological/enzymatic activity. Therefore, warmer hives can maximize task performance, such as the evaporation of water from nectar [65], wax production [81], or larval growth rates [82]. Irrespective of the reasons for these daily temperature cycles, any deviations from these daily cycles could provide early indicators of climatic stress, which should be studied in more detail in the future. The temperature and humidity ranges we calculated differed from those provided in the literature [29,58,59,60,61,65,70,71,72,83]. This implies that absolute measures of hive humidity and temperature depend on additional factors, such as bee genotype, type of housing, or the climatic conditions in the environment. Similar to what we already mentioned earlier, ranges than absolute measures such as means seem better measures to define and understand colony homeostasis.

## Figures and Tables

**Table 1:** An overview of temperature and humidity measures obtained from honey bee hives kept in Warré and Langstroth setups. Mean and median temperatures (in °C), relative (in %), and absolute humidity (in g/m^3^) ± 95% CI are provided for both hive types as well as for the mornings and afternoons.

Variable	Hive Type	Time of the Day		
			9 a.m.	3 p.m.
Temperature (°C)	Langstroth	Mean ± 95% CI Median ± 95% CI	31.00 ± 0.30 31.00 [31.00, 31.50]	33.90 ± 0.20 34.50 [34.00, 34.50]
		Minimum	19.00	25.00
		Maximum	36.50	39.00
	Warré	Mean ± 95% CI Median ± 95% CI	31.60 ± 0.30 32.00 [31.50, 32.50]	33.70 ± 0.20 34.00 [34.00, 34.50]
		Minimum	20.50	26.00
		Maximum	37.00	40.50
Relative Humidity (RH in %)	Langstroth	Mean ± 95% CI Median ± 95% CI	53.80 ± 0.90 52.50 [52.00, 54.00]	49.1 ± 0.90 49.5 [48.00, 50.50]
		Minimum	34.00	24.50
		Maximum	85.00	75.50
	Warré	Mean ± 95% CI Median ± 95% CI	50.20 ± 0.90 50.50 [48.50, 52.00]	43.40 ± 0.80 43.00 [41.50, 44.00]
		Minimum	28.00	24.00
		Maximum	77.50	74.00
Absolute Humidity (AH in g/m^3^)	Langstroth	Mean ± 95% CI Median ± 95% CI	17.25 ± 0.30 16.98 [16.70, 17.50]	18.30 ± 0.30 18.40 [17.90, 18.70]
		Minimum	9.80	10.40
		Maximum	27.20	27.60
	Warré	Mean ± 95% CI Median ± 95% CI	16.60 ± 0.30 16.20 [15.90, 16.40]	16.07 ± 0.20 15.85 [15.70, 16.20]
		Minimum	9.60	9.80
		Maximum	30.90	27.10

## Data Availability

The data are available upon request from the corresponding author B.B.
